# Renin-Angiotensin System overactivation in polycystic ovary syndrome, a risk for SARS-CoV-2 infection?

**DOI:** 10.1016/j.metop.2020.100052

**Published:** 2020-08-18

**Authors:** Abu Saleh Md Moin, Thozhukat Sathyapalan, Stephen L. Atkin, Alexandra E. Butler

**Affiliations:** a Diabetes Research Center (DRC), Qatar Biomedical Research Institute (QBRI), Hamad Bin Khalifa University (HBKU), Qatar Foundation (QF), PO Box 34110, Doha, Qatar; b Academic Endocrinology, Diabetes and Metabolism, Hull York Medical School, Hull, UK; c Royal College of Surgeons in Ireland Bahrain, Adliya, Bahrain

**Keywords:** ACE2 protein, Polycystic ovary syndrome, Renin, Angiotensinogen

## Abstract

**Background:**

The SARS-CoV-2 coronavirus gains entry to target cells via the angiotensin-converting enzyme 2 (ACE2) receptor present on cells in blood vessels, lungs, heart, intestines, and kidneys. Renin-Angiotensin System (RAS) overactivity has also been described in metabolic syndrome, type 2 diabetes (T2D) and obesity, conditions shared by women with polycystic ovary syndrome (PCOS) We hypothesized that RAS overactivity may be present in PCOS.

**Methods:**

We determined plasma levels of RAS-related proteins in a cohort of age matched control women (n = 97) and women with PCOS (n = 146). Plasma levels of RAS-related proteins (ACE2, Renin and Angiotensinogen (AGT)) were determined by Slow Off-rate Modified Aptamer (SOMA)-scan plasma protein measurement.

**Results:**

PCOS women had a higher BMI (p < 0.001), systolic (p < 0.0001) and diastolic (p < 0.05) blood pressure, waist circumference (p < 0.0001), testosterone (p < 0.0001), free androgen index (p < 0.0001) and CRP (p < 0.0001). Renin was elevated in PCOS (p < 0.05) and angiotensinogen was lower in PCOS (p < 0.05), indicating overactivity of the RAS system in PCOS. ACE2 levels were lower in PCOS (p < 0.05), suggesting that PCOS women are at risk for development of hypertension.

**Conclusion:**

RAS proteins levels differed between PCOS and control women, suggesting that the insulin resistance inherent in PCOS may predispose these women to more severe COVID-19 infection.

## Highlights

• Renin-Angiotensin System (RAS) overactivity has been described in Type 2 diabetes (T2D) and obesity.
• These conditions represent high risk for COVID-19 infection and severe disease.
• Renin-Angiotensin System overactivation is present in PCOS and is independent of obesity.
• Women with PCOS may be predisposed to more severe COVID-19 infection.

## 1 Background

The SARS-CoV-2 coronavirus gains entry to target cells via the angiotensin-converting enzyme 2 (ACE2) receptor present on cells in blood vessels, lungs, heart, intestines, and kidneys.

The Renin-Angiotensin System (RAS) system plays a major role in blood pressure regulation, via regulation of sodium and water balance, and RAS overactivation is an established risk factor for development of renal [1] and cardiovascular disease [2]. RAS overactivity has also been described in metabolic syndrome [3], type 2 diabetes (T2D) and obesity [4], all high risk conditions for COVID-19 infection and severe disease.

In vitro studies have found that hyperglycemia stimulates the RAS system at the tissue level [[5], [6], [7], [8], [9], [10]] and tissue RAS activity may be a central facet in metabolic disorders [11].

ACE2 is one of the arms of the RAS, located on the X chromosome [12,13]. ACE2 is a monocarboxypeptidase that cleaves various substrates [12,14,15] including an octapeptide angiotensin II (Ang-II) to generate Ang-(1–7), a biologically active metabolite of the RAS which acts on the membrane bound MasR (Mas receptor) [16]. This ‘ACE2/Ang-(1–7)/Mas’ axis serves as a protective arm of RAS by providing the physiological antagonism of its well-established classical ACE/AngII/AT1R system [16,17].

ACE2 has been shown to play a beneficial role in the pathophysiology of diabetes [18,19] and its related complications [[20], [21], [22], [23]]. The emerging evidence suggests that the modulation of the ACE2/Ang-(1–7)/Mas receptor axis is a very attractive target in the therapy of the metabolic syndrome and diabetes-associated diseases affecting the heart and the kidney. For example, activators of endogenous ACE2 such as xanthenone and diminazene aceturate have been identified as compounds capable of increasing ACE2 activity to counteract the overactive RAS [24]. ACE2 activators appear to be beneficial in attenuating hyperglycemia as well as diabetic complications such as hypertension and endothelial dysfunction in diabetic subjects [21,24,25]. Several meta-analyses have underscored the positive effects of ARBs (angiotensin receptor blockers) and ACE inhibitors on insulin sensitivity and the progression to Type 2 diabetes [26,27]. Since the ACE2/Ang-(1–7)/Mas receptor axis naturally counterbalances the effects of classical RAS components, it is reasonable to believe that part of the positive effects of ARBs and ACE inhibitors on metabolic diseases could be mediated by overactivation of the Ang-(1–7) pathway. Indeed, a recent study has suggested that the beneficial effects of olmesartan, an ARB, on vascular remodeling are mediated via activation of the ACE2/Ang-(1–7)/Mas receptor axis [28]. Thus, ACE2 activators provide a novel avenue to control T2DM and related complications.

Most of the available studies have shown that diabetes mellitus (DM) as a distinctive comorbidity is associated with more severe COVID-19 disease, acute respiratory distress syndrome and increased mortality [[29], [30], [31]]. Hyperglycemia and a diagnosis of T2DM were also independent predictors of mortality and morbidity in patients with SARS [32]. In addition, critically ill patients with COVID-19 have been reported to be in an extreme hypermetabolic state [33].

Women with polycystic ovary syndrome share features of metabolic syndrome, including insulin resistance [34] and obesity [35], with a high proportion going on to develop type 2 diabetes [36]. Therefore, the cardio-metabolic diseases commonly seen in women with PCOS overlap with risk factors predisposing to severe COVID-19 disease [37]. Further, case reports of pregnant woman with polycystic ovary syndrome (PCOS) infected by SARS-CoV-2 [38] suggest PCOS as a high-risk factor for COVID-19.

## 2 Objective

We hypothesized that RAS overactivity may also be present in conditions of insulin resistance, such as PCOS; we therefore determined levels of RAS-related proteins in a cohort of age matched women with and without PCOS.

## 3 Methods

146 PCOS and 97 control women who presented sequentially to the Department of Endocrinology, Hull and East Yorkshire Hospitals NHS Trust were recruited to the local PCOS biobank (ISRCTN70196169). The Newcastle & North Tyneside Ethics committee approved this study; all patients gave written informed consent. PCOS diagnosis was based on all three Rotterdam consensus diagnostic criteria; all fulfilled NIH criteria. None were taking hormone replacement therapy.

Following plasma collection, circulating levels of RAS-related proteins (ACE2, Renin and Angiotensinogen (AGT)) were determined by Slow Off-rate Modified Aptamer (SOMA)-scan plasma protein measurement [39]. Statistics were performed using Graphpad Prism 8.0.

## 4 Results

**Fig. 1:**
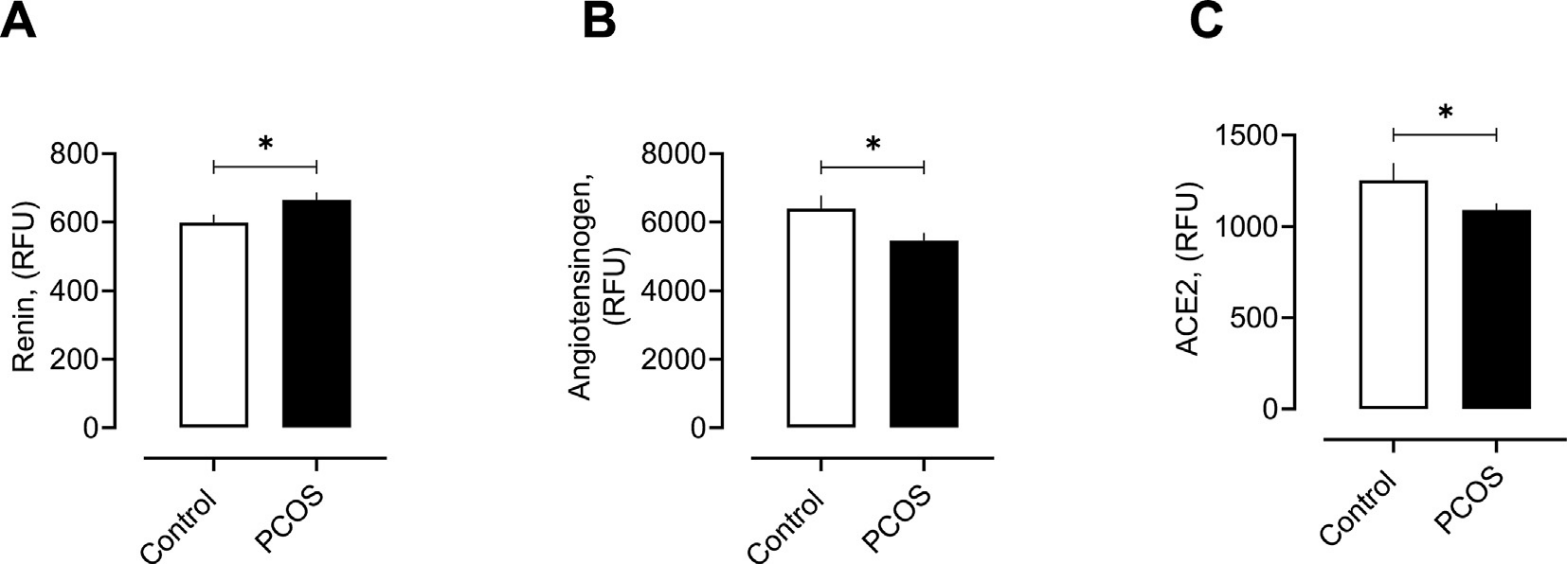
**RAS proteins in women with and without polycystic ovary syndrome (PCOS)**. Levels of plasma Renin (A), Angiotensinogen (B) and ACE2 (C) in women with and without polycystic ovary syndrome (PCOS). RFU, relative fluorescent units. ∗p < 0.05.

While the cohorts were matched for age, PCOS women had a higher BMI (p < 0.001), systolic (p < 0.0001) and diastolic (p < 0.05) blood pressure, waist circumference (p < 0.0001), testosterone (p < 0.0001), free androgen index (p < 0.0001) and CRP (p < 0.0001). Circulatory renin was elevated in PCOS (665 ± 22 vs 600 ± 23 RFU, PCOS vs control, p < 0.05) and angiotensinogen was low in PCOS (5368 ± 213 vs 6394 ± 383 RFU, PCOS vs control, p < 0.05), indicating overactivity of the RAS system in PCOS (Fig. 1). ACE2 levels were lower in PCOS (1090 ± 37 vs 1253 ± 95 RFU, PCOS vs control, p < 0.05) (Fig. 1), suggesting that PCOS women are at risk for development of hypertension. No relationship was found with BMI, systolic or diastolic blood pressure and any RAS-related proteins measured here.

## 5 Discussion

This study showing elevated plasma renin, together with suppressed angiotensinogen and reduced levels of ACE2 protein suggests RAS overactivation in PCOS occurs in an obesity-independent manner.

Renin induces conversion of angiotensinogen to angiotensin I (ANGI); ANGI is then converted to ANGII by ACE. In contrast, ACE2 converts ANGII to ANG-1-7, a normotension-maintenance peptide. Reduced ACE2 levels may predispose to increased severity of COVID-19 infection. Pulmonary ACE2 has been shown to protect against lung injury and the loss of ACE2 in acute lung injury results in leakage from pulmonary blood vessels mediated by angiotensin I receptor stimulation [40]. Further, disruption of the RAS system is associated with pulmonary hypertension and fibrosis [40]. Angiotensin II upregulates the expression of profibrotic cytokines leading to pulmonary fibrosis and severe inflammation with increased vascular permeability, a scenario that may be attenuated by angiotensinogen converting enzyme (ACE) inhibitors and angiotensin receptor blocker (ARB) therapies [40]. In addition, PCOS women might also be vulnerable to COVID-19 because of their high androgen levels, as reported here. Binding of androgen to androgen receptor elements (AREs) regulates transcription of transmembrane serine protease 2 (TMPRSS2) [41] that activates the SARS-Cov-2 spike protein, facilitating viral entry. The combination of an overactive RAS system (androgen-independent) together with increased androgens places women with PCOS at increased risk for serious COVID-19 infection.

**Fig. 2:**
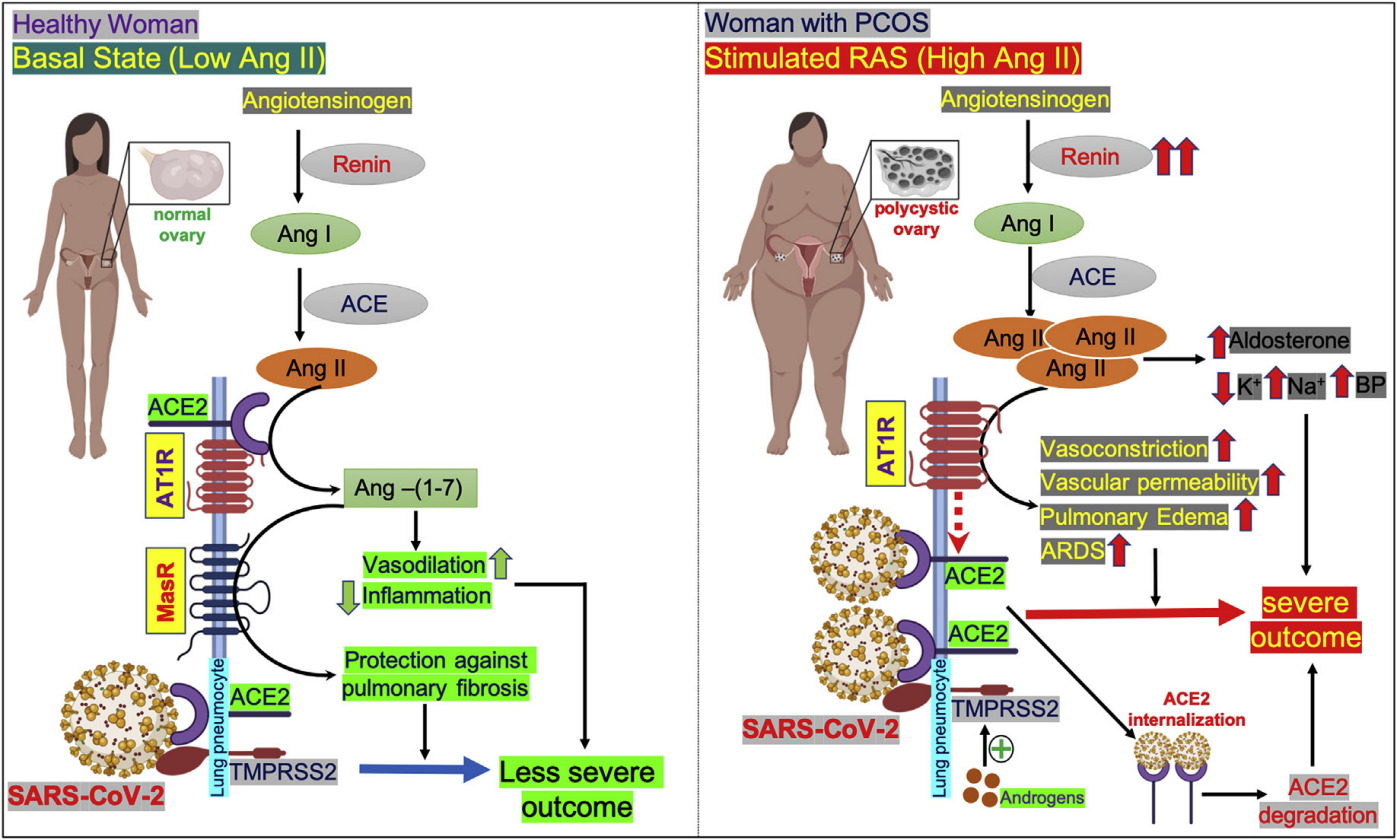
**Schematic diagram of proposed mechanism of COVID-19 severity in women with polycystic ovary syndrome (PCOS)**. ***Left panel***, in health, under normal physiological conditions (basal state), angiotensinogen is converted into angiotensin I (Ang I) by the hormone/enzyme renin. Ang I is further converted into angiotensin II (Ang II) with the help of the enzyme angiotensin converting enzyme (ACE). Ang II binds to its receptors, angiotensin receptor 1 (AT1R) or angiotensin converting enzyme 2 (ACE2), in different cell surfaces. At basal state, when the Ang II level is low in lung pneumocytes, the catalytic site of the AT1R is occupied with ACE2; therefore, Ang II cannot interact with AT1R and it is then hydrolyzed by ACE2 into angiotensin (1–7) [Ang- (1–7)]. Ang-(1–7) has a cardioprotective effect as it is a potent vasodilator and reduces inflammation. In lung, Ang-(1–7) binds to the Mas receptor (MasR) and protects lung pneumocytes from pulmonary fibrosis. ACE2 serves as the receptor for SARS-CoV-2 and, with the help of a serine protease TMPRSS2, it infects the lung pneumocytes. Since the Ang II level is low in basal conditions, ACE2 remains attached to AT1R and, therefore, there is less access for SARS-CoV-2 to bind to its receptors, resulting in less severe impact in COVID-19. ***Right panel***, in polycystic ovary syndrome (PCOS) women, the plasma renin level is high and the RAS is overactivated, leading to the production of high amounts of Ang II. Excess Ang II causes the dissociation of ACE2 from AT1R and binds to AT1R. Binding of Ang II to AT1R results in vasoconstriction, increased vascular permeability, pulmonary edema and ARDS. When ACE2 becomes detached from AT1R (indicated by broken red arrow), it increases the entry point for SARS-CoV-2 into lung pneumocytes. The viral infection might also be facilitated by overexpression of androgen-induced expression of TMPRSS2 in PCOS, as the androgen levels are higher in PCOS. Upon binding with ACE2, the SARS-CoV-2 -ACE2 complex becomes internalized and undergoes proteasomal degradation of ACE2 inside the cell. This may also cause the reduction of ACE2 levels in lung cells. High Ang II levels also stimulates the adrenal gland to increase aldosterone level which, in turn, decreases potassium and increases sodium levels, and ultimately causes increased blood pressure. Taken together, all these mechanisms could result in a severe outcome for COVID-19-infected women with PCOS. (For interpretation of the references to colour in this figure legend, the reader is referred to the Web version of this article.)

The possible mechanism of overactivated RAS in severe outcome of COVID-19 in women with PCOS has been outlined in Fig. 2.

Limitations of this study include (1) measurement of plasma proteins that may not be reflective of tissue levels and (2) measurement of renin concentrations rather than activity.

In conclusion, RAS protein levels differed between PCOS and control women, suggesting that the insulin resistance inherent in PCOS may predispose these women to more severe COVID-19 infection.

## Ethics approval and consent to participate

The Newcastle & North Tyneside Ethics committee approved this study. All patients gave written informed consent.

## Consent for publication

All authors gave their consent for publication.

## Availability of data and materials

All the data for this study will be made available upon reasonable request to the corresponding author.

## Funding

No funding was received to perform this study.

## Author contributions

ASMM and AEB analyzed the data and wrote the manuscript. TS supervised clinical studies and edited the manuscript. SLA contributed to study design, data interpretation and the writing of the manuscript. All authors reviewed and approved the final version of the manuscript. Alexandra E Butler is the guarantor of this work.

## CRediT authorship contribution statement

**Abu Saleh Md Moin:** data analysis, Writing - original draft, Writing - review & editing, All authors reviewed and approved the final version of the manuscript. **Thozhukat Sathyapalan:** Supervision, clinical studies, Writing - review & editing, All authors reviewed and approved the final version of the manuscript. **Stephen L. Atkin:** Conceptualization, Writing - review & editing, Data interpretation. All authors reviewed and approved the final version of the manuscript. **Alexandra E. Butler:** data analysis, Writing - original draft, Writing - review & editing, guarantor of this work. All authors reviewed and approved the final version of the manuscript.

## Declaration of competing interest

No authors have any conflict of interest or competing interests to declare.
